# One-Pot Synthesis Method of MIL-96 Monolith and Its CO_2_ Adsorption Performance

**DOI:** 10.1021/acsami.2c22955

**Published:** 2023-05-01

**Authors:** Motomu Sakai, Hayata Hori, Takaya Matsumoto, Masahiko Matsukata

**Affiliations:** † Research Organization for Nano & Life Innovation, Waseda University, 513 Wasedatsurumaki-cho, Shinjuku-ku, Tokyo 162-0041, Japan; ‡ Department of Applied Chemistry, Waseda University, 513 Wasedatsurumaki-cho, Shinjuku-ku, Tokyo 162-0041, Japan; § Central Technical Research Laboratory, ENEOS Corporation, 8 Chidoricho, Naka-ku, Yokohama, Kanagawa 231-0815, Japan; ∥ Advanced Research Institute for Science and Engineering, Waseda University, 3-4-1 Okubo, Shinjuku-ku, Tokyo 169-0085, Japan

**Keywords:** metal−organic framework, monolith, adsorption, CO_2_, MIL-96

## Abstract

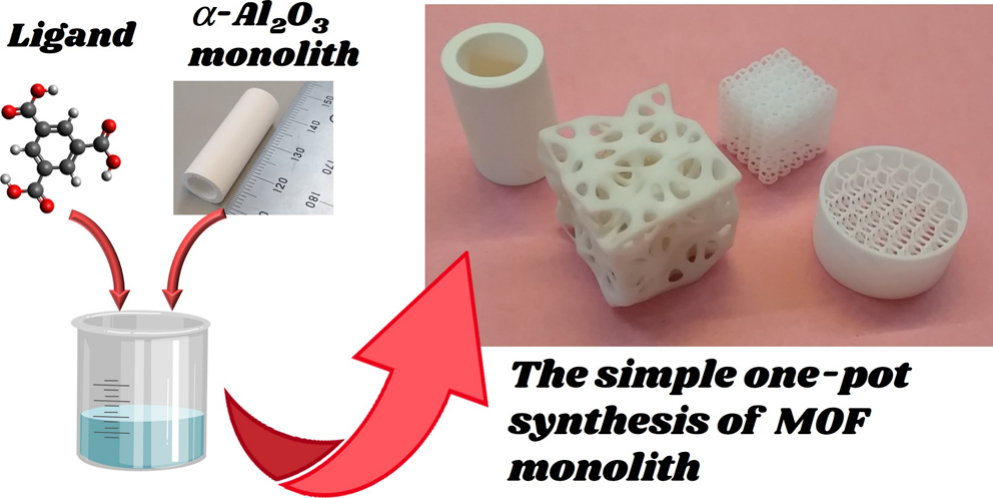

A novel preparation method was proposed for a metal–organic framework (MOF) monolith using a simple one-pot synthesis method. A MOF tubular monolith was successfully prepared by the hydrothermal treatment for an α-Al_2_O_3_ monolith in an aqueous solution of 1,3,5-benzenetricarboxylic acid and nitric acid without the addition of a metal source. The effects of temperature and the HNO_3_ concentration in the synthesis solution on the crystallization behavior of MIL-96 were studied. HNO_3_ enhanced the dissolution of the α-Al_2_O_3_ monolith and the growth of MIL-96. The growth rate of MIL-96 was also influenced by the synthesis temperature; a synthesis temperature of over 453 K was required for crystallization. The CO_2_ adsorption capacity of the prepared MIL-96 monoliths was evaluated and found to be comparable to that of the well-grown MIL-96 powdery crystal. Furthermore, the MIL-96 monoliths demonstrated good stability as their adsorption properties were retained even after 2 months of storage under atmospheric conditions.

## 1 Introduction

Carbon capture, utilization, and storage (CCUS) technologies have been widely studied for implementing carbon neutrality. The bottleneck in CCUS technology is the high cost of the carbon capture step, which consumes more than 60% of the entire CCS process cost.^1,2^ Therefore, a CO_2_ separation technology with low energy consumption is highly desirable.

Adsorption separation using solid adsorbents is a promising technique for CO_2_ capture with low energy consumption. Various solid materials for CO_2_ capture have been studied in recent decades, including carbon-based materials, silica, alumina, zeolites, polymers, and metal–organic frameworks (MOFs).^3^

MOFs, also known as porous coordination polymers, have attracted considerable attention as novel adsorbents. MOFs are porous materials comprising metals and organic ligands. By tuning the combination of metal and ligand species, the pore sizes, pore networks, and adsorption properties of the MOF can be controlled. A wide variety of MOFs with excellent CO_2_ adsorption properties have been developed.^4,5^

MIL-96 is a MOF composed of Al and trimesic acid (TMA, 1,3,5-benzenetricarboxylic acid). MIL-96 has attracted attention as a novel CO_2_ adsorbent because of its high CO_2_ adsorption capacity and high resistance to humidity and high temperature.^6−8^ Owing to its high stability, MIL-96 is also expected to be an adsorbent for a wide range of applications, such as the separation of light hydrocarbons,^9^ the capture of iodine in organics,^10^ and the defluoridation of water.^11^

Despite many interesting studies on MOF synthesis, there are a few examples of the industrial application of MOFs as adsorbents. Although an adsorbent should be granulated for use in the adsorption process, MOF crystals can easily break into tiny particles of fine powder, and adsorbent loss and pipe clogging inevitably occur.^12^ Hence, several articles and reviews have highlighted the need to shape MOF powders into millimeter-sized objects with sufficient mechanical strength for their effective use in potential applications.^12−14^ Therefore, progress in shaping techniques is crucial for the utilization of MOFs as adsorbents.

Shape-forming technologies for MOFs have also been reported, including processing methods such as pressing or extrusion under high pressure with binders.^12,15^ Whereas pressing is one of the easiest methods to obtain MOF pellets, unfortunately, the use of inactive binders and the associated pore blockage can result in pellets with low porosity and adsorption capacity. Bazer-Bachi et al. studied the effect of compression on the adsorption properties of MOFs (ZIF-8, HKUST-1, and SIM-1) and reported irreversible changes due to the loss of crystallinity in proportion to the applied force.^13^

MOFs are often mixed with substrates such as polymers or aerogels for shaping.^16,17^ Zhang et al. prepared a filter by processing a MOF into a nanofibrous filter,^16^ which effectively adsorbed toxic gases, such as SO_2_.

Recently, a new preparation method was developed to obtain MOF monoliths. Several strategies have been reported for the synthesis of MOF monoliths, including (1) coating the MOF on a preshaped monolith and growth, (2) shaping a mixture of a MOF and binder into a monolith, (3) shaping a mixture of a binder and metal source and/or organic ligand into a monolith and crystallizing it into a MOF monolith, (4) crystallizing a metal monolith into a MOF monolith, and (5) crystallization of a metal oxide into a MOF monolith. For example, in case 1, Ramos-Fernandez et al. prepared a MIL-101(Cr) monolith by seeding and secondary growth. In this study, a seed crystal of MIL-101(Cr) was loaded onto a cordierite monolith and grown in a synthesis solution.^18^ In case 2, Kaskel et al. reported a Cu_3_(BTC)_2_ monolith prepared via the extrusion of a mixture of the MOF and binder.^19^ In case 3, Rezaei et al. reported shaping a mixture of MOF precursors and binders (kaolin or bentonite) into a honeycomb monolith by 3D printing, followed by hydrothermal or solvothermal treatment of the preshaped monolith to obtain an MOF monolith.^20,21^ Kim et al. developed a growth method for MOFs (MIL-53, HKUST-1, and ZIF-7) on metal fibers and meshes,^22^ using them as both substrates and metal sources for MOFs. MOF monoliths derived from metal oxide monoliths have rarely been reported. Liang et al. reported the preparation of MOF monoliths (MIL-53(Al), HKUST-1, ZIF-8, and ZIF-67) from metal oxide sheets prepared through electrospinning of sol–gel precursors followed by calcination.^23^ These methods described above have enabled the production of MOF monoliths with complex shapes and large surface areas.

For MOF monoliths, it is very important to achieve both strength and adsorption properties. Using a metal oxide substrate for monoliths has several advantages, including expanded thermal and chemical stabilities of the substrate, and a variety of choices of atomic species and shapes for ceramic monoliths. In this paper, we propose a novel technique for preparing MIL-96 monoliths using a commercially available α-Al_2_O_3_ monolith. Our approach is to convert an α-Al_2_O_3_ monolith into an MIL-96 monolith directly via a simple one-pot synthesis in an aqueous solution of 1,3,5-benzenetricarboxylic acid without addition of a metal source. This method does not require pretreatment of the monoliths, such as seeding or surface modification with organic ligands. This technique allows for the easy production of MOF monoliths with practical sizes and mechanical strengths. We investigated the formation process of a MIL-96 monolith using this method and evaluated the CO_2_ adsorption property of the MIL-96 monolith, comparing it with that of MIL-96 powdery crystals. The mechanical strength of the MIL-96 monolith was also evaluated.

## 2 Experimental Section

### 2.1 Materials and Chemicals

An α-Al_2_O_3_ tubular monolith (length = 10 mm, inner diameter = 7 mm, and outer diameter = 10 mm) was used to prepare the MIL-96 monolith. The monolith was washed with distilled water and acetone through sonication. After washing, the monolith was dried at 373 K before use.

Nitric acid (HNO_3_, Kanto Chemical), 1,3,5-benzenetricarboxylic acid (trimesic acid, TMA, Sigma-Aldrich), and distilled water were used for the synthesis. Further purification of the chemicals was not performed before use.

### 2.2 MOF Monolith Preparation

HNO_3_, TMA, and tubular α-Al_2_O_3_ were used as the raw materials for preparing the MIL-96 monolith. Subsequently, 0.654 g of TMA was added to 37.5 g of distilled water. The mixture was stirred at 353 K until TMA was completely dissolved. After adding a certain amount of HNO_3_, the mixture was poured into a glass container with 1.5 g of α-Al_2_O_3_ tubular monolith. The concentration of HNO_3_ in the synthesis solution was varied from 0 to 0.7 M. The glass container was placed in a stainless-steel autoclave and sealed. Hydrothermal treatment was performed at a certain temperature and for a given period (423–473 K, 1–14 days). After the hydrothermal treatment, the autoclave was quenched with flowing tap water to stop the reaction. The monolith and precipitated powders were separately obtained via filtration. Both the monolith and the powder were washed with ethanol and distilled water and dried at 383 K.

The prepared monolith was named *M*–*X*–*Y–Z*, where *X*, *Y*, and *Z* represent the synthesis temperature [K], HNO_3_ concentration [M], and synthesis period [days], respectively.

MIL-96 powder was synthesized as a reference material according to the literature.^6^ A mixture of TMA, aluminum nitrate nonahydrate, and distilled water was hydrothermally treated at 453 K for 24 h. The detail of the preparation procedure is provided in Supporting Information.

### 2.3 Calculation of Al Conversion

The Al conversion was determined to understand the formation process of MIL-96 from the α-Al_2_O_3_ monolith as follows.

In this study, a portion of the α-Al_2_O_3_ monolith was consumed through three pathways: dissolution into an aqueous solution, crystallization of the precipitated MIL-96 powdery crystals, and crystallization of MIL-96 on the monolith. Consequently, we procured the monolith, synthesis solution, and precipitated powder after the reaction. Here, we defined the number of Al atoms in the raw Al_2_O_3_ monolith as *A* [mol], that dissolved in the solution as *B* [mol], that in the precipitate as *C* [mol], and that converted to MIL-96 in the monolith as *D* [mol]. The weight change of the entire monolith before and after synthesis was defined as Δ*W* [g].

The number of Al atoms in the raw Al_2_O_3_ monolith, *A*, was calculated based on the weight of the monolith before synthesis. The number of Al dissolved in aqueous solution, *B*, was evaluated using inductively coupled plasma (ARCOS, SPECTRO Analytical Instruments). The concentration of Al in the reaction solution was measured, and the amount of dissolved Al was calculated. In addition, the number of Al in the precipitated powder, *C*, was measured using thermogravimetric (TG) analysis. The precipitated powder was calcined in flowing air using TG, and the amount of Al remaining after calcination in the Al_2_O_3_ form was determined.

During the synthesis, the weight of the entire monolith was reduced through the leaching of Al and increased by capturing organic ligands with the formation of the MOF. Thus, the weight change Δ*W* [g] of the entire monolith can be expressed using *A*–*D* as follows
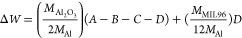
1where *M*_Al_2_O_3__, *M*_Al_, and *M*_MIL96_ represent the molecular and atomic weights of Al_2_O_3_, Al atoms, and MIL-96, respectively. In this study, these values were 101.96, 26.98, and 1962.86 g mol^–1^, respectively (calculated from Al_12_O–(OH)_16_(H_2_O)_5_[BTC]_6_).^7^ The first and second terms on the right side of eq 1 indicate the weight reduction due to Al leaching and weight gain due to the capture of organic ligands during the formation of the MOF, respectively. Using this equation, we calculated the amount of Al converted to the MIL-96 in monolith *D*.

### 2.4 CO_2_ Adsorption Test

The CO_2_ adsorption properties of the prepared MOF powders and monoliths were evaluated using the volumetric adsorption method (Belsorp-MAX, MicrotracBEL). The equipment had a special sample holder that allowed the insertion of an entire monolith without cutting or fracturing. The detailed structure of the sample holder is described elsewhere.^24^ CO_2_ adsorption tests were performed at 283–333 K for the samples, which were pretreated at 423 K for 8 h under vacuum. To calculate the amount of adsorbed CO_2_ on the monolith, the weight of MIL-96 in the monolith was used rather than the entire weight of the monolith.

### 2.5 Cleavage Test for the α-Al_2_O_3_ Tube and MIL-96 Monolith

A cleavage test was conducted to evaluate the mechanical strength of the MOF monoliths. An α-Al_2_O_3_ tubular monolith or MIL-96 monolith lying horizontally was sandwiched between two plates, and the applied load at which the monolith fractured was recorded. A tensile testing machine (AG-I 250 kN, Shimadzu) equipped with a load cell of 10 kN was used for testing. The samples were pressed at a rate of 1 mm min^–1^.

## 3 Results and Discussion

### 3.1 Effect of HNO_3_ Concentration on MIL-96 Crystallization Behavior

The HNO_3_ concentration was varied from 0 to 0.7 M, and its effect on the MIL-96 crystallization behavior was studied. MIL-96 monoliths were hydrothermally grown in aqueous TMA at 453 K for 1–7 days with different concentrations of HNO_3_.

**Figure 1:**
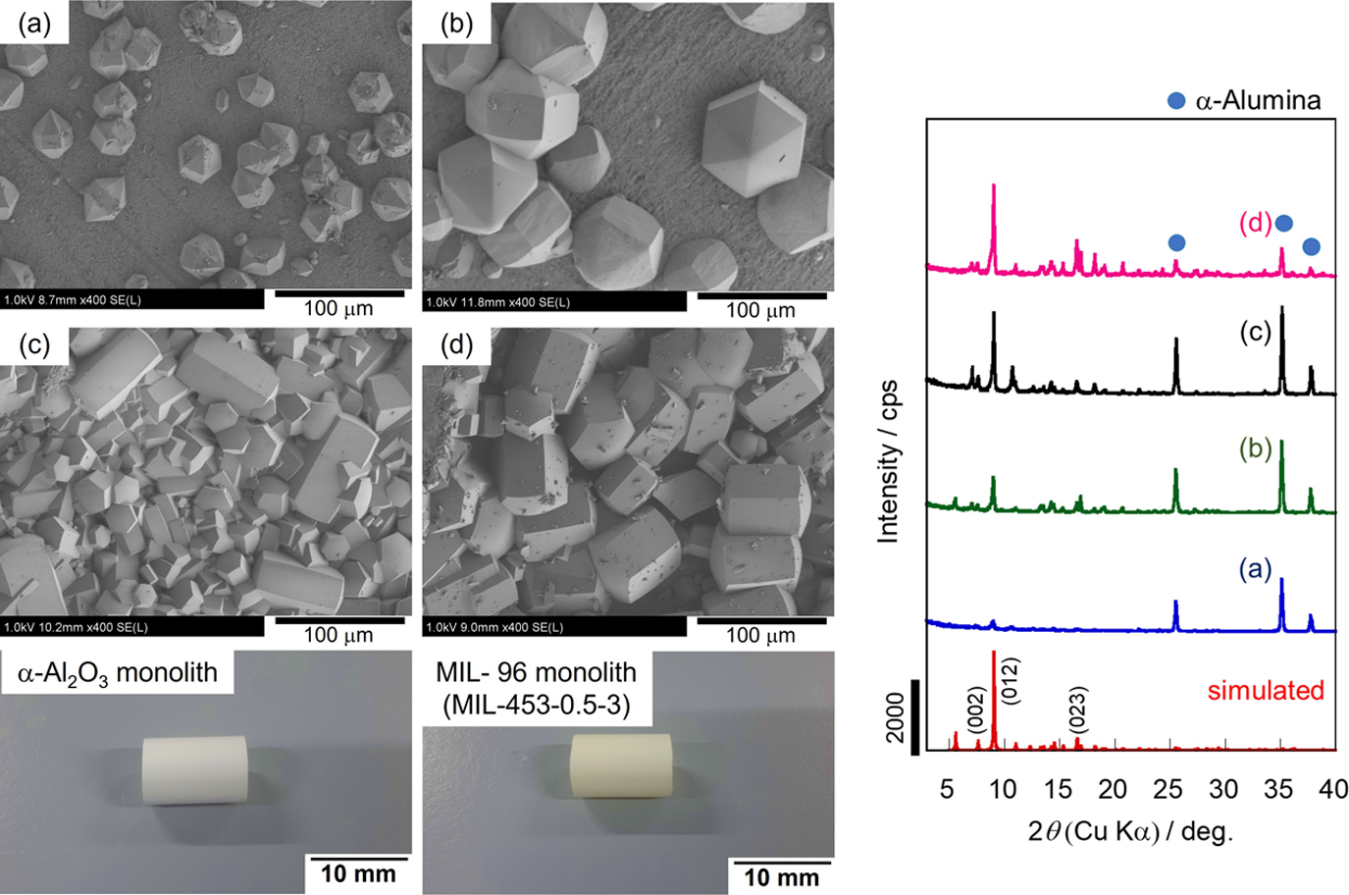
XRD patterns and typical FE-SEM images of the monoliths synthesized at 453 K for 3 days with (a) 0, (b) 0.1, (c) 0.5, and (d) 0.7 M HNO_3_ aq. The pictures show the α-Al_2_O_3_ monolith and *M*-453-0.5-3.

Figure 1 shows the typical FE-SEM images and XRD patterns of the monolith *M*-453-*Y*-3 after crystallization for 3 days with different HNO_3_ concentrations. Images of the α-Al_2_O_3_ monolith and *M*-453-0.5-3 are also shown.

The FE-SEM images show that the surfaces of the α-Al_2_O_3_ monolith were entirely covered with MIL-96 crystals at higher HNO_3_ concentrations (0.5 and 0.7 M). The inner surface was also converted to MIL-96 under these conditions. In addition, the typical diffraction patterns of the MIL-96 crystal and α-Al_2_O_3_ were observed in all the samples, based on the XRD results. These results clearly show that the α-Al_2_O_3_ monolith was successfully converted into the MIL-96 monolith in this simple one-pot synthesis without the addition of a metal source.

The HNO_3_ concentration affected the crystal morphology, that is, the hexagonal bipyramidal shape and the XRD pattern. At higher HNO_3_ concentrations, the lengths of the hexagonal columns between the hexagonal pyramids increased. This result agrees with the relationship between the acid concentration and crystal morphology reported by Liu et al.^25^ The intensity ratio of the (002) to (012) peaks in the prepared monoliths was changed by varying the HNO_3_ concentration. The intensity of the (002) peak is strongly affected by the crystal morphology.^7^ This peak appears prominently in plate-like crystals and is hardly observed in hexagonal rod crystals. As shown in the SEM images, the MIL-96 crystals in the monolith grew as hexagonal rods with higher HNO_3_ concentrations, resulting in relatively weakened diffraction derived from (002).

**Figure 2:**
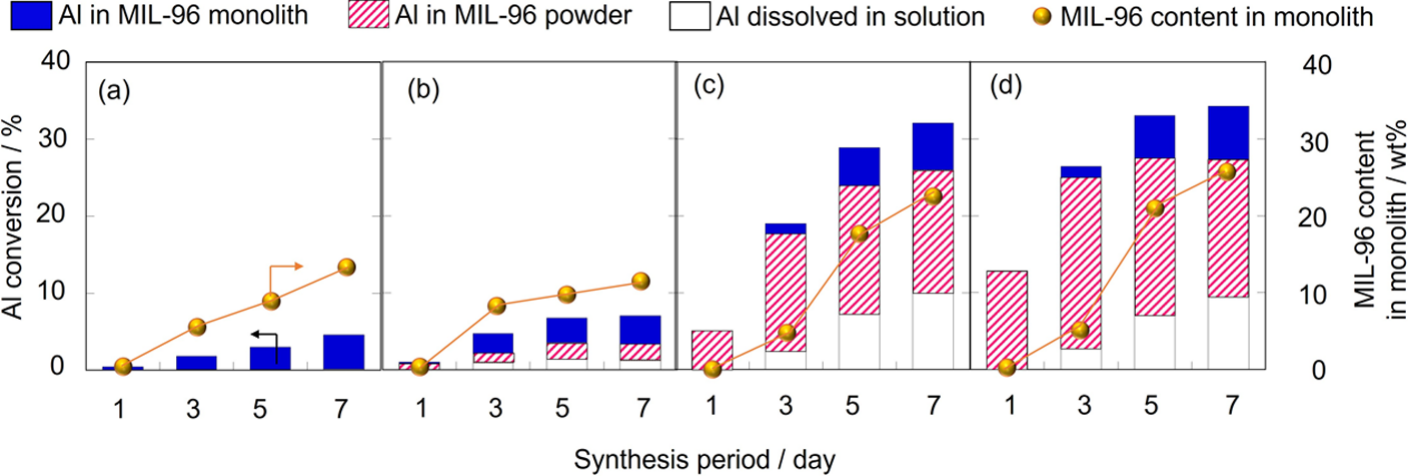
Consumption of Al in the α-Al_2_O_3_ monolith synthesized at 453 K with (a) 0, (b) 0.1, (c) 0.5, and (d) 0.7 M HNO_3_ aq. The solid and shaded bars show the conversion of Al to the MIL-96 monolith and powdery crystal. The open bar represents the amount of Al consumed for dissolution. The circle plot shows the MIL-96 content in the monolith.

Figure 2 depicts the change in Al consumption in the α-Al_2_O_3_ monolith in the course of synthesis. At low HNO_3_ concentrations, MIL-96 was slowly formed in the α-Al_2_O_3_ monolith, leading to an increase in the conversion of Al to the MIL-96 monolith from 0.1 to 3.7 mol % in 0.1 M of HNO_3_ aq with increasing synthesis period. Higher HNO_3_ concentrations resulted in a faster growth of MIL-96 in the monolith, with the conversion of Al in the monolith to MIL-96 reaching 6.5 mol % after 7 days of crystallization with 0.7 M aqueous HNO_3_. In this case, the weight of the MIL-96 crystal in the monolith was 23 wt %.

The addition of aqueous HNO_3_ caused the leaching of Al from the α-Al_2_O_3_ monolith. This phenomenon was not observed in the absence of HNO_3_. MIL-96 powdery crystals precipitated as Al dissolved. The HNO_3_ concentration significantly influenced the amount of Al dissolved from the monolith and precipitated MIL-96 powder. For example, 18 mol % Al dissolved in the solution, and 9.5 mol % Al was converted into MIL-96 powdery crystals.

Here, we discuss the effect of HNO_3_ on the crystallization of MIL-96. The MIL-96 monolith was obtained by hydrothermal treatment of the α-Al_2_O_3_ monolith in TMA aq without HNO_3_ aq, suggesting that Al in the α-Al_2_O_3_ monolith directly reacted with TMA and formed MIL-96 crystals. In contrast, the addition of HNO_3_ aq increased both the number of MIL-96 crystals in the monolith and in the precipitate, indicating that the Al species dissolved from α-Al_2_O_3_ monolith facilitated the nucleation and/or crystal growth of MIL-96 in both the monolith and the bulk solution. Although the addition of HNO_3_ was not essential for the formation of the MIL-96 monolith, the growth of MIL-96 in the monolith was promoted by the addition of HNO_3_.

### 3.2 Influence of Synthesis Temperature on MIL-96 Growth

The effect of synthesis temperature was studied. Hydrothermal treatment was carried out in the range of 423–473 K in TMA with 0.5 M HNO_3_.

**Figure 3:**
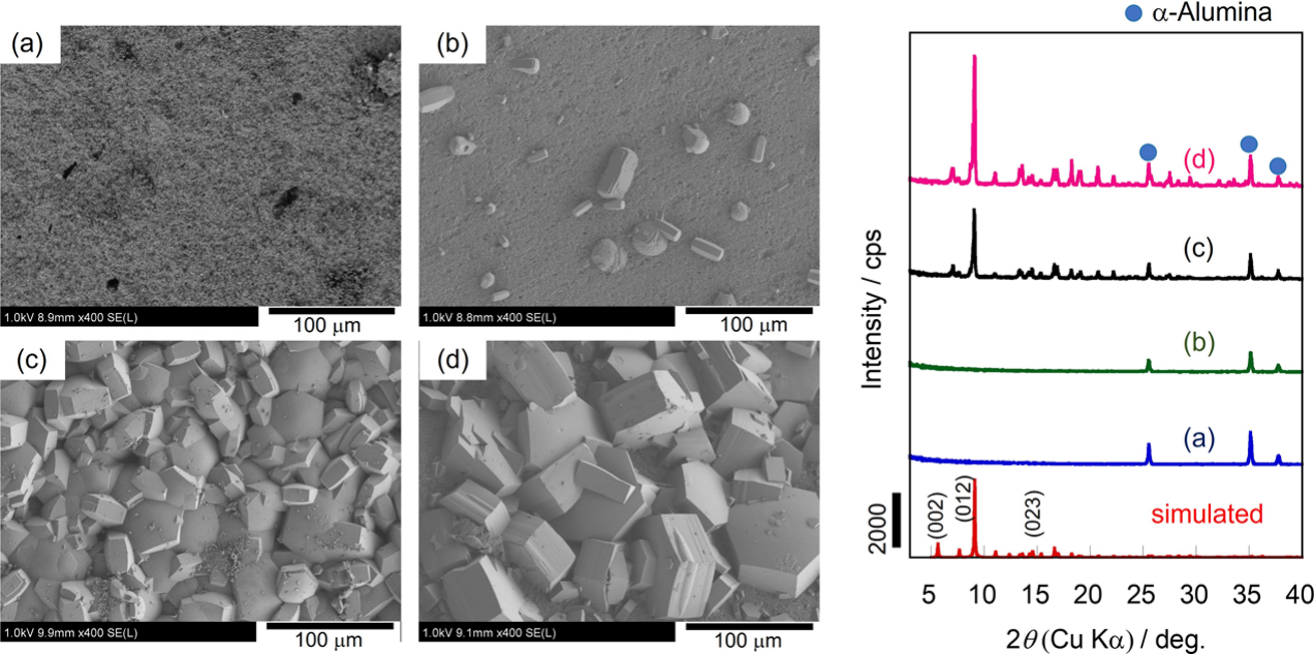
XRD patterns and typical FE-SEM images of the surface of the monoliths synthesized at (a) 423, (b) 443, (c) 463, and (d) 473 K for 3 days with 0.5 M HNO_3_ aq.

Figure 3 shows typical FE-SEM images and XRD patterns of the monolith *M*-*X*-0.5-3. Very few MIL-96 crystals were observed on the surface of the monolith grown below 443 K. The XRD results were consistent with the FE-SEM images.

At higher temperatures, the size of MIL-96 crystals increased, while the number of crystals observed on the monolith surface decreased. This result indicates that MIL-96 crystals grew through the Ostwald ripening, where crystals grew from the surrounding ones, thereby decreasing their total number. Crystal growth on a substrate in the Ostwald ripening mode has been previously reported for ZIF-8.^26^

**Figure 4:**
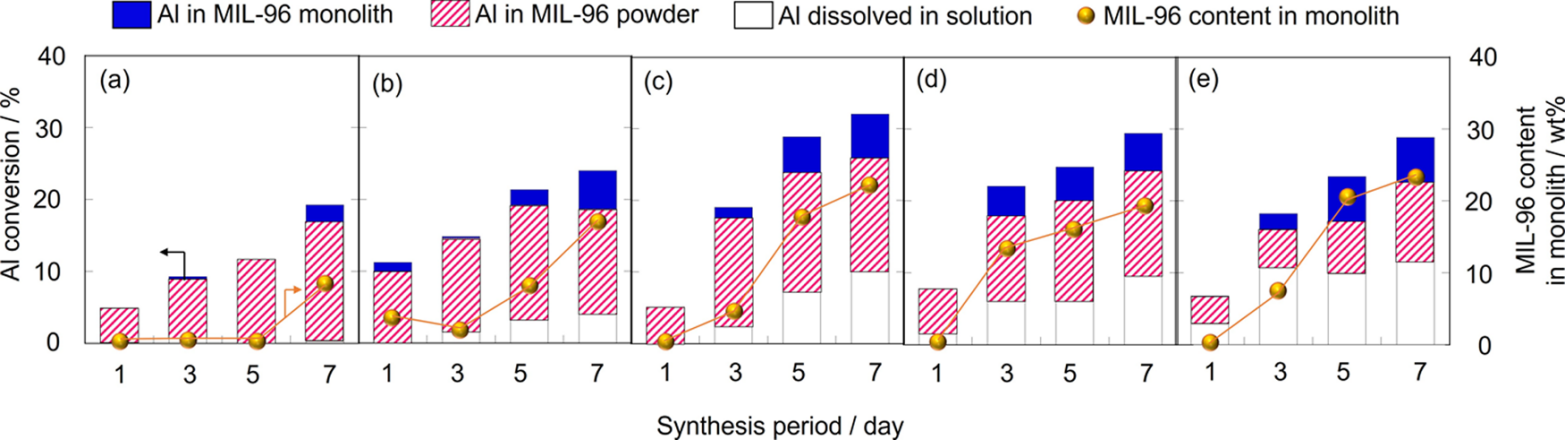
Consumption of Al in the α-Al_2_O_3_ monolith synthesized at (a) 423, (b) 443, (c) 453, (d) 463, and (e) 473 K with 0.5 M HNO_3_ aq. The solid and shaded bars show the conversion of Al to the MIL-96 monolith and powdery crystal. The open bar represents the amount of Al consumed for dissolution. The circle plot shows the MIL-96 content in the monolith.

The results presented in Figure 4 demonstrate the temperature effect of Al consumption in the α-Al_2_O_3_ monolith. At 423 K, MIL-96 crystals were hardly generated in either the monolith or the precipitate, consistent with the results shown in Figure 3. At 443 K, a small number of MIL-96 crystals were formed in the monolith. The growth rate of MIL-96 remarkably increased above 453 K, leading to the Al conversion to the MIL-96 monolith reaching 6.2 mol % (21 wt %). The number of precipitated powdery crystals increased with increasing synthesis temperature, possibly due to a large amount of Al dissolved in the solution contributing to the nucleation and growth of MIL-96 powdery crystals in the bulk solution.

A large number of previous studies reported that MIL-96 was synthesized at temperatures above 473 K and was hardly obtained at lower temperatures.^6−8^ The fact that crystallization did not proceed below 443 K is not a specific phenomenon in monolith preparation but rather a general trend in MIL-96 synthesis.

**Figure 5:**
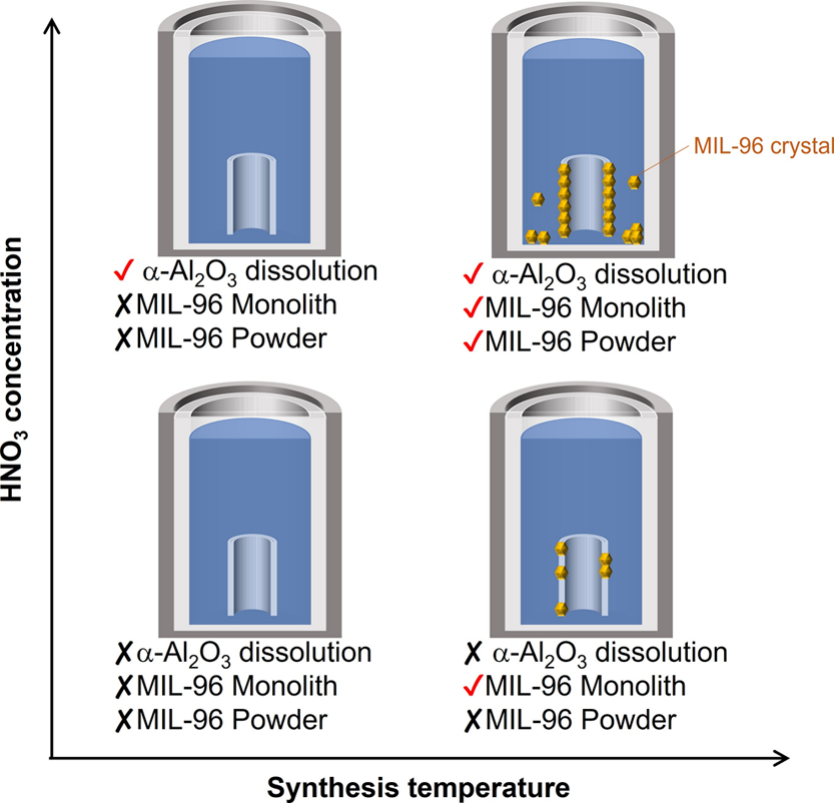
Schematic illustration of the effect of HNO_3_ concentration and synthesis temperature on crystallization behavior.

Figure 5 shows a schematic illustration of the effect of HNO_3_ concentration and synthesis temperature on the crystallization behavior, as indicated by the results shown in Figures 1–4.

This novel synthetic method was demonstrated for an α-Al_2_O_3_ monolith with a more complex shape. We successfully confirmed the direct conversion of the porous α-Al_2_O_3_ filter, as shown in Figure S1, although the Al conversion of the MIL-96 monolith was small. The mechanical strength of the MIL-96 monoliths was also evaluated. Table 1 lists the loads applied when the monolith was fractured in a cleavage test. Although the mechanical strength of MIL-96 monoliths was lower than that of α-Al_2_O_3_ monoliths, MIL-96 monoliths had sufficient mechanical strength to serve as adsorbents. Figure S2 presents images of the cleavage test.

**Table 1:** Results of Cleavage Tests

	load when monolith fractured/N		
sample	run 1	run 2	run 3
α-Al_2_O_3_	155.75	196.75	183.65
*M*-453-0.5-3	112.85	138.75	125.35
*M*-453-0.5-7	69.20	85.05	109.40

### 3.3 Adsorption Behavior of the MIL-96 Monolith

We evaluated the adsorption properties of MIL-96 monoliths and compared them with those of MIL-96 powder. CO_2_ adsorption tests were conducted after pretreatment at 423 K for 8 h under vacuum. In the case of the MIL-96 monolith, the weight of MIL-96 generated was used as the denominator to calculate the amount adsorbed. The amount adsorbed per monolith weight is listed in the Supporting Information (Table S1).

**Figure 6:**
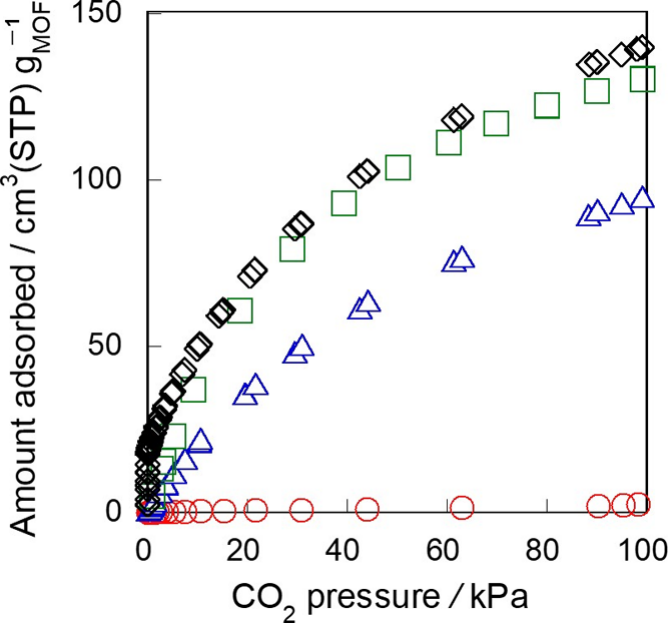
Adsorption isotherms of CO_2_ on MIL-96 monoliths and powder at 283 K. ○, *M*-453-0-3; Δ, *M*-453-0.5-3; □, *M*-473-0.5-3; ◊, powder.

CO_2_ adsorption tests were performed on three types of monoliths, *M*-453-0-3, *M*-453-0.5-3, *M*-473-0.5-3, and powdery crystals. Figure 6 shows the CO_2_ adsorption isotherms on MIL-96 monoliths and powder at 283 K. The amount of CO_2_ adsorbed on each monolith significantly depended on the synthesis conditions. The adsorbed amounts on *M*-453-0-3, *M*-453-0.5-3, and *M*-473-0.5-3 at 100 kPa were 2.34, 86.8, and 130 cm^3^ (STP) g^–1^, respectively. For comparison purposes, the amount of CO_2_ adsorbed on MIL-96 powder was measured and found to be 140 cm^3^ (STP) g^–1^ at 100 kPa, which almost agreed with the values reported in the previous study.^8^

*M*-453-0-3, prepared in the absence of HNO_3_, showed very low CO_2_ adsorption, suggesting that the micropore of *M*-453-0-3 was almost completely plugged by unreacted materials such as TMA. In contrast, *M*-453-0.5-3 and *M*-473-0.5-3, obtained in the presence of HNO_3_, exhibited relatively high CO_2_ adsorption capacities. *M*-473-0.5-3 exhibits the highest CO_2_ adsorption capacity among the three monoliths, which can be attributed to the increase in crystallinity at the high synthesis temperature. Notably, the amount of adsorbed CO_2_ on *M*-473-0.5-3 was comparable to that adsorbed on MIL-96 powder.

It is well known that the CO_2_ adsorption capacity of MIL-96 is often affected by the synthesis method and conditions. Table 2 presents the amounts of CO_2_ adsorbed on MIL-96 synthesized using various methods and conditions.^6−8,25,27^ This table clearly shows that *M*-473-0.5-3 grew well and had a good CO_2_ adsorption capacity.

**Table 2:** CO_2_ Adsorbed Amounts of MIL-96 Powders and the Monolith

entry	adsorption temperature/K	CO_2_ amount adsorbed at 100 kPa/cm^3^ g^–1^	references
MIL-96 powder	303	73	(6)
MIL-96 powder	273	108	(7)
MIL-96 powder	273	124	(8)
MIL-96 powder	298	84	(25)
MIL-96 powder	303	61	(27)
MIL-96 monolith	298	105	this study

**Figure 7:**
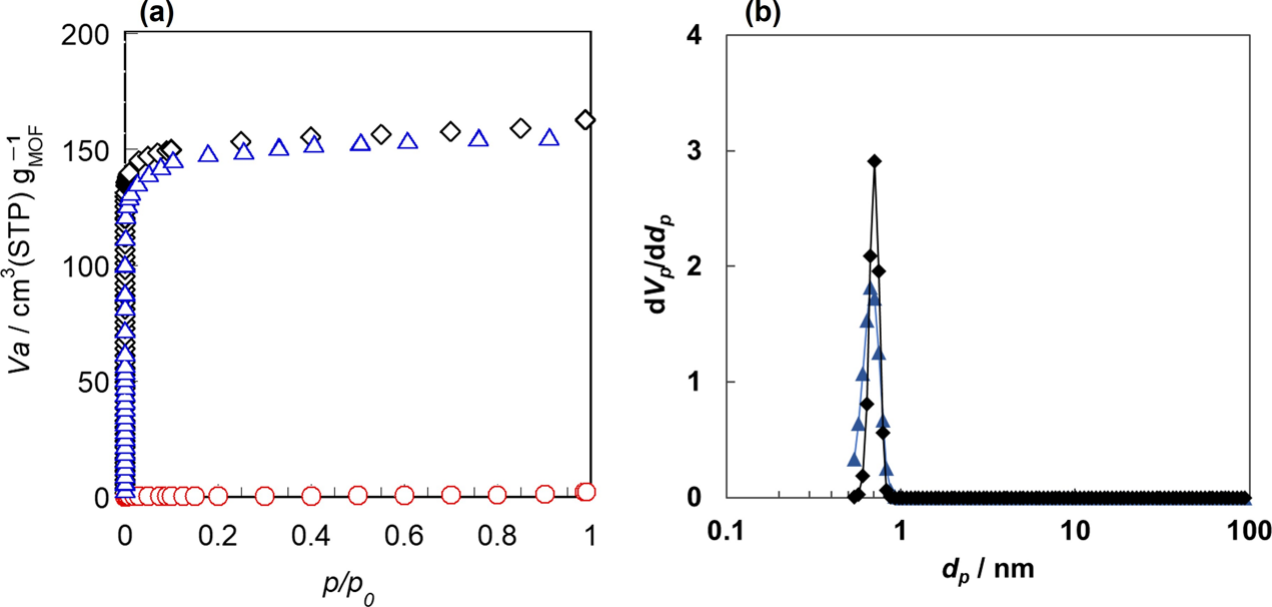
(a) Adsorption isotherms of N_2_ on MIL-96 monoliths and powder at 77 K. (b) Pore size distribution calculated assuming cylindrical pores by GCMC. ○, *M*-453-0-3; Δ, *M*-473-0.5-3; ◊, powder.

Figure 7 shows the N_2_ isotherms and pore size distribution obtained for MIL-96 monoliths and powder at 77 K. As is the case of CO_2_ adsorption, the weight of MIL-96 generated was used as the denominator for the calculation of the amount adsorbed. *M*-453-0-3 showed almost zero N_2_ adsorption capacity even at 77 K, indicating that it was a nonporous material. Moreover, the amount of N_2_ adsorbed on *M*-473-0.5-3 was slightly lower than that adsorbed on MIL-96 powder. These results are consistent with the CO_2_ adsorption results (Figure 6), supporting that the CO_2_ adsorption capacity of the MIL-96 monolith can be explained by the micropore volume. Moreover, MIL-96 powder and monolith had almost the same pore size distribution calculated assuming cylindrical pores by GCMC.

**Figure 8:**
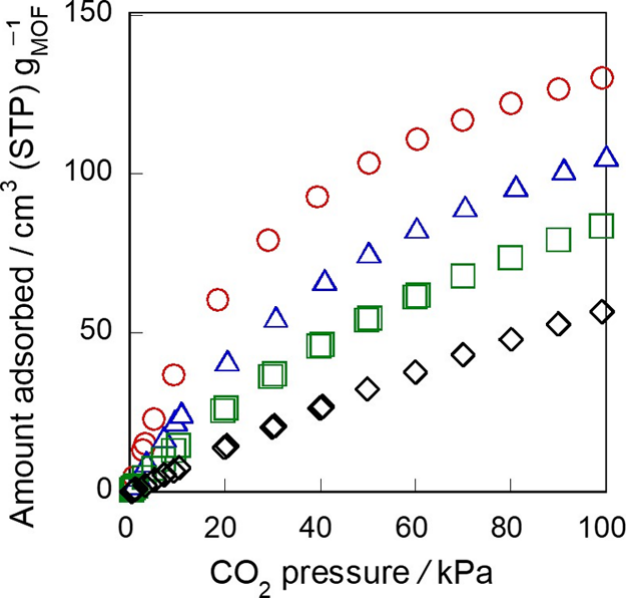
Adsorption isotherms of CO_2_ on *M*-473-0.5-3 at (○) 283, (Δ) 298, (□) 313, and (◊) 333 K.

Figure 8 shows the adsorption isotherms of CO_2_ on *M*-473-0.5-3 at 283–333 K. Unsurprisingly, the amount of adsorbed CO_2_ tends to be smaller at higher temperatures. Specifically, CO_2_ adsorbed amounts at 100 kPa and at 283 and 333 K were 130 and 58 cm^3^ (STP) g^–1^, respectively. This result suggests that the MIL-96 monolith prepared in this study had a large working capacity around ambient temperature and was suitable for temperature-swing adsorption of CO_2_.

**Figure 9:**
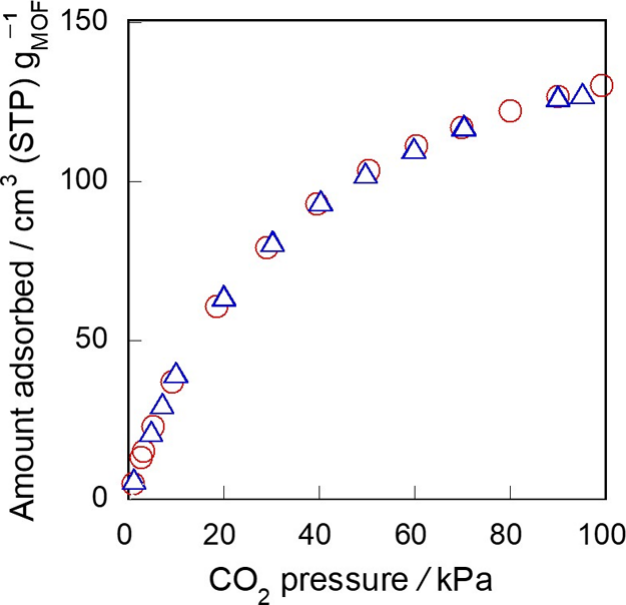
Comparison of the adsorption isotherms of CO_2_ on *M*-473-0.5-3 (○) before and (Δ) after the storage in air for 2 months.

The stability of the MIL-96 monolith in air was also studied. Figure 9 shows the CO_2_ adsorption isotherms on fresh *M*-473-0.5-3 and the sample stored in air for 2 months. The adsorption isotherm obtained for the stored sample was in good agreement with that for the fresh sample, indicating that the monolith was highly stable in air and humidity.

## 4 Conclusions

Hydrothermal treatment of the α-Al_2_O_3_ monolith in an aqueous solution of TMA and HNO_3_ provided a simple one-pot synthesis route of the MIL-96 monolith without the addition of a metal source. In this method, α-Al_2_O_3_ was directly converted to MIL-96. The synthesis conditions, such as the synthesis temperature and HNO_3_ concentration in the synthesis solution, significantly affected the crystallization behavior of MIL-96.

The monolith exhibited a good CO_2_ adsorption capacity, almost the same as that of the well-crystallized MIL-96 powder, and maintained its adsorption property for CO_2_ even after 2 months while also exhibiting good mechanical strength.

This simple one-pot synthesis enabled us to convert complex-shaped monoliths, such as formed or 3D-printed ceramics, resulting in MOF adsorbents with high surface areas and low flow resistances, making them promising candidates in industrial applications.

## Supporting Information Available

Pictures of the MIL-96 monolith converted from the porous α-Al_2_O_3_ filter and the equipment for the cleavage test (PDF)
