# Supplementary material for: One-Pot Synthesis Method of MIL-96 Monolith and Its CO2 Adsorption Performance
Source: ACS Appl Mater Interfaces. 2023 May 1;15(18):22395–402. doi: 10.1021/acsami.2c22955 (PMC10176467; doi:10.1021/acsami.2c22955)
Supplement: Supplementary file 1 — am2c22955_si_001.pdf [file am2c22955_si_001.pdf]

Supporting information

One-pot Synthesis Method of MIL-96 Monolith and its CO_2 Adsorption Performance

Motomu Sakai[*,1], *Hayata Hori*[2], *Takaya Matsumoto*[3], *and Masahiko Matsukata*[1,2,4]

[1]Research Organization for Nano & Life Innovation, Waseda University, 513 Wasedatsurumaki-cho, Shinjuku-ku, Tokyo 162-0041, Japan

[2]Department of Applied Chemistry, Waseda University, 513 Wasedatsurumaki-cho, Shinjuku-ku, Tokyo 162-0041, Japan

[3]ENEOS corporation, 1-1-2 Otemachi, Chiyoda-ku, Tokyo 100-8162, Japan.

[4]Advanced Research Institute for Science and Engineering, Waseda University, 3-4-1 Okubo, Shinjuku-ku, Tokyo 169-0085, Japan

*saka.moto@aoni.waseda.jp

Preparation procedure of MIL-96 powdery crystal

MIL-96 powdery crystal was synthesized by hydrothermal treatment according to previous report[1]. Ligand solution was prepared by mixing of 1.1 g of trimesic acid and 20 g of distilled water. 9.4 g of aluminium nitrate nonahydrate was added into 30 g of distilled water for the preparing of metal source. Ligand solution and metal source solution was vigorously mixed for 10 min, and then the mixture was poured into Teflon-lined autoclave. Hydrothermal treatment was carried out at 453 K for 24 h in an air oven. After crystallization, precipitated powder was obtained by filtration and washed with the mixture of ethanol and distilled water. The powder was dried at 343 K in atmosphere.

MIL-96 monolith synthesis from porous α-Al_2O_3 monolith

The novel one-pot synthesis was applied for α-Al_2O_3 monolith with complex shape. The synthesis was performed at 453 K for 3 days in TMA aq with 0.5 M of HNO_3 aq. Fig. S1 shows the pictures of tubular monolith and porous filter.

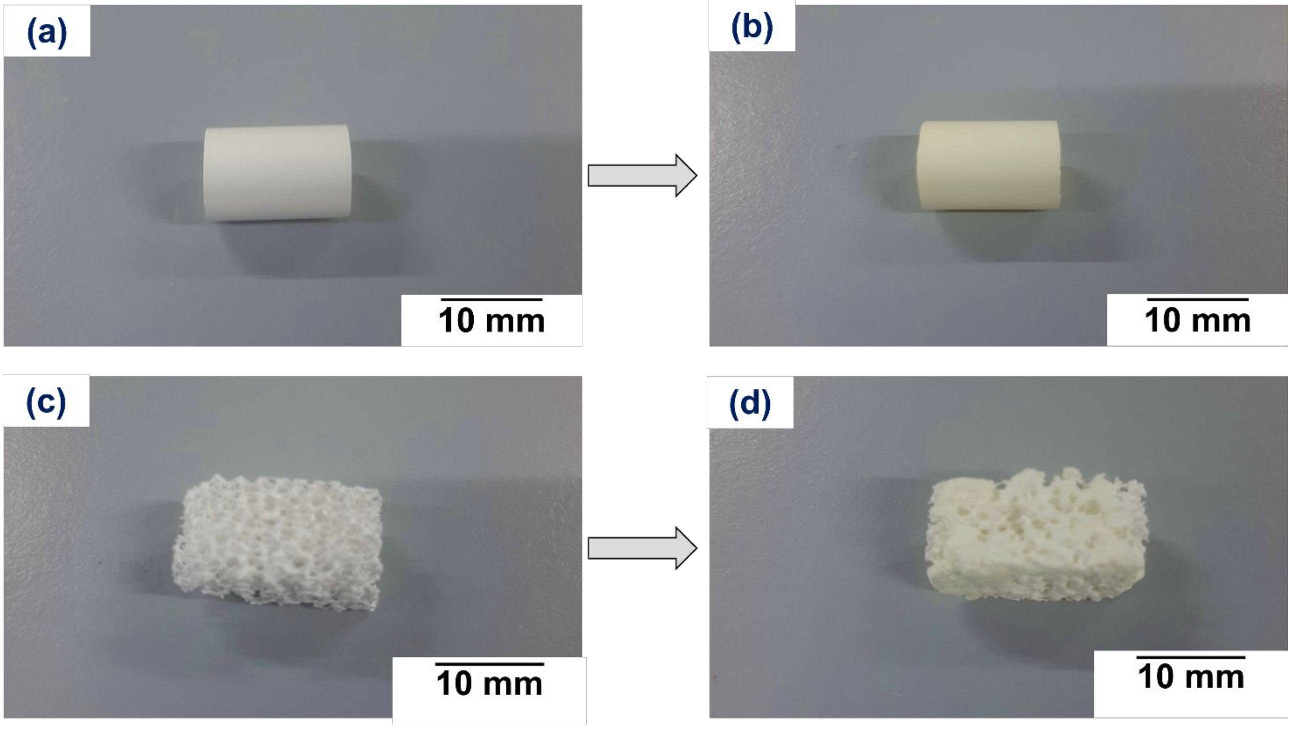

Figure S1 The pictures of (a) tubular α-Al_2O_3, (b) tubular MIL-96 monolith (M-453-0.5-3), (c) porous α-Al_2O_3 filter, (d) porous MIL-96 monolith.

Cleavage test

A cleavage test was performed to evaluate the mechanical strength of MOF monolith. An α-Al_2O_3 tubular monolith or MIL-96 monolith laid horizontally was sandwiched between two plates, and the load when the monolith broke was recorded. Fig. S2 shows the photographs of the test.

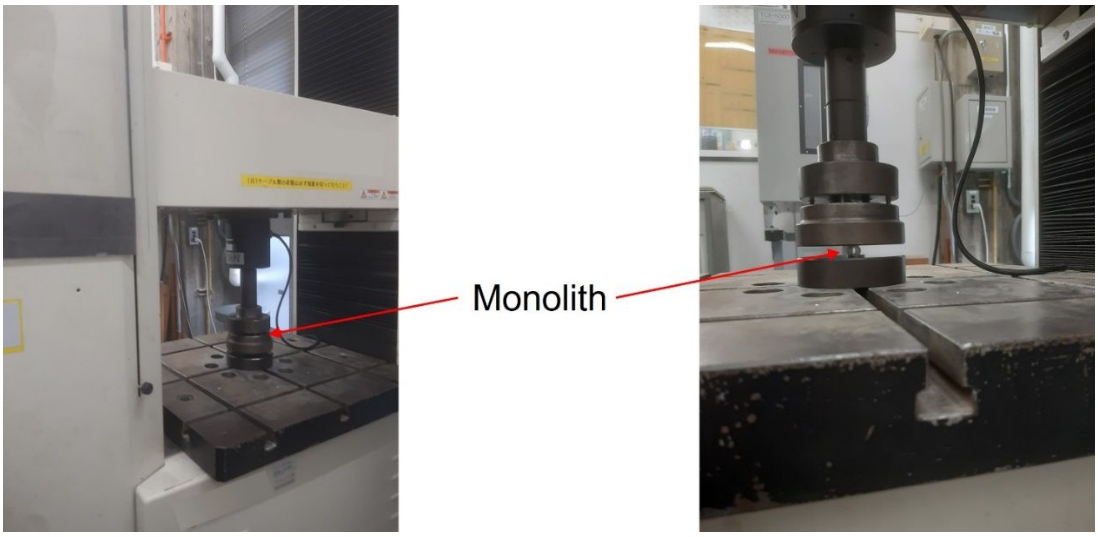

Figure S2 The pictures the cleavage test.

Amount adsorbed per monolith weight

Table S1 lists the amount adsorbed of N_2 and CO_2 on MIL-96 powder and monoliths. Both adsorption amount per MOF weight and monolith weight were provided for comparison.

Table S1 Amount adsorbed of N_2 and CO_2 on MIL-96 powder and monoliths

Sample	Amount adsorbed / cm^3(STP) g_{MOF}^{-1}		Amount adsorbed / cm^3(STP) $g_{monolith}^{-1}$	
	N_2	CO_2	N_2	CO_2
powder	162.7 at 77 K	140.1 at 283 K	N/A	N/A
M-453-0-3	2.38 at 77 K	2.34 at 283 K	0.14 at 77 K	0.14 at 283 K

M-453-0.5-3	N/A	86.8 at 283 K	N/A	4.34 at 283 K
		130.3 at 283 K		10.4 at 283 K
M-473-0.5-3	155.4 at 77 K	105.7 at 298 K	12.4 at 77 K	8.24 at 283 K
		83.6 at 313 K		6.69 at 283 K
		57.8 at 333 K		4.62 at 283 K
M-473-0.5-3 stored in air for 2 months	N/A	127.5 at 283 K	N/A	10.2 at 283 K
M-473-0.5-7	N/A	119.1 at 283 K	N/A	28.6 at 283 K

[1] Benoit, V.; Chanut, N.; Pillai, R. S.; Benzaqui, M.; Beurroies, I.; Devautour-Vinot, S.; Serre, C.; Steunou, N.; Maurin, G.; Llewellyn, P. L. A Promising Metal-Organic Framework (MOF), MIL-96(Al), for CO2 separation under humid conditions. J. Mater. Chem. A 2018, 6, 2081–2090.
